# Crystal structure of Hg_2_SO_4_ – a redetermination

**DOI:** 10.1107/S1600536814011155

**Published:** 2014-08-01

**Authors:** Matthias Weil

**Affiliations:** a Institute for Chemical Technologies and Analytics, Division of Structural Chemistry, Vienna University of Technology, Getreidemarkt 9/164-SC, A-1060 Vienna, Austria

**Keywords:** crystal structure, redetermination, mercurous, Hg/S/O system

## Abstract

The crystal structure of mercury(I) sulfate (or mercurous sulfate), Hg_2_SO_4_, was re-determined based on modern CCD data. In comparison with the previous determination from Weissenberg film data [Dorm (1969 ▶). *Acta Chem. Scand.* **23**, 1607–1615], all atoms were refined with anisotropic displacement parameters, leading to higher precision in terms of bond lengths and angles [*e.g*. Hg—Hg = 2.5031 (7) compared to 2.500 (3)Å]. The structure consists of alternating rows along [001] of Hg_2_^2+^ dumbbells (generated by inversion symmetry) and SO_4_^2−^ tetra­hedra (symmetry 2). The dumbbells are linked *via* short O—Hg—Hg—O bonds to the sulfate tetra­hedra into chains extending parallel to [20-1]. More remote O—Hg—Hg—O bonds connect these chains into a three-dimensional framework.

## Related literature   

Structural data of the previous refinement of Hg_2_SO_4_ (Dorm, 1969 ▶) were deposited with the ICSD (2014 ▶), but contain an error in the *z* coordinate of the sulfur atom. Other phases in the system Hg/S/O were structurally characterized by Aurivillius & Stålhandske (1980 ▶) [HgSO_4_], Weil (2001 ▶) [Hg_3_(SO_4_)O_2_] and Logemann & Wickleder (2013 ▶) [Hg(S_2_O_7_)]. For a review on Hg—Hg bond lengths in mercurous compounds, see: Weil *et al.* (2005 ▶).

## Experimental   

### Crystal data  

Hg_2_O_4_S
*M* *_r_* = 497.24
Monoclinic, 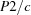

*a* = 6.2771 (8) Å
*b* = 4.4290 (6) Å
*c* = 8.3596 (10) Å
β = 91.695 (4)°
*V* = 232.31 (5) Å^3^
*Z* = 2
Mo *K*α radiation
μ = 66.35 mm^−1^
*T* = 295 K
0.18 × 0.08 × 0.04 mm

### Data collection  

Bruker SMART CCD diffractometer
Absorption correction: numerical (*HABITUS*; Herrendorf, 1997 ▶) *T*_min_ = 0.012, *T*_max_ = 0.119
1737 measured reflections
701 independent reflections
629 reflections with *I* > 2σ(*I*)’
*R*_int_ = 0.060

### Refinement  

*R*[*F* ^2^ > 2σ(*F* ^2^)] = 0.035
*wR*(*F* ^2^) = 0.087
*S* = 1.06
701 reflections
34 parameters
Δρ_max_ = 3.57 e Å^−3^
Δρ_min_ = −3.18 e Å^−3^

### 

Data collection: *SMART* (Bruker, 2005 ▶); cell refinement: *SAINT* (Bruker, 2005 ▶); data reduction: *SAINT*; program(s) used to solve structure: *SHELXS97* (Sheldrick, 2008 ▶); program(s) used to refine structure: *SHELXL97* (Sheldrick, 2008 ▶); molecular graphics: *ATOMS for Windows* (Dowty, 2006 ▶); software used to prepare material for publication: *publCIF* (Westrip, 2010 ▶).

## Supplementary Material

Crystal structure: contains datablock(s) I, global. DOI: 10.1107/S1600536814011155/hb0012sup1.cif

Structure factors: contains datablock(s) I. DOI: 10.1107/S1600536814011155/hb0012Isup2.hkl

**Fig. 1.**
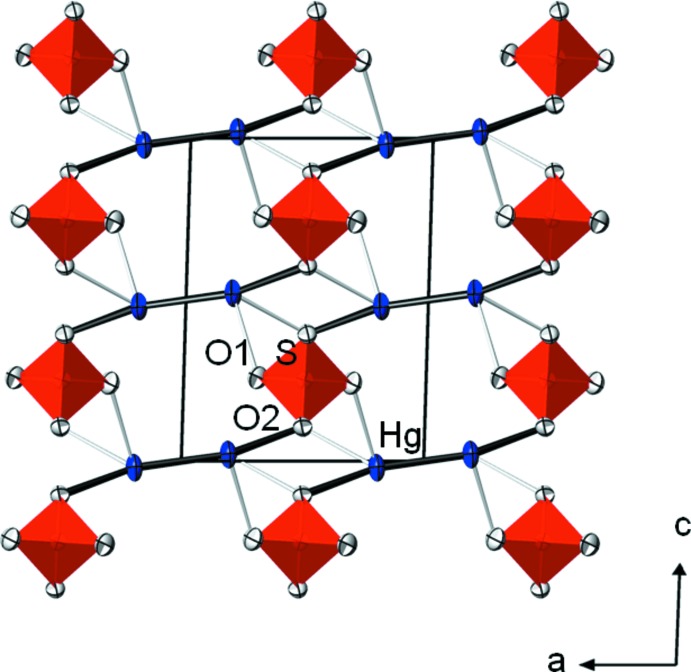
The crystal structure of Hg2SO4 in a projection along [010]. Displacement ellipsoids are drawn at the 74% probability level; short Hg—O bonds are displayed with closed black lines, longer Hg—O bonds with open lines.

2 4 . DOI: 10.1107/S1600536814011155/hb0012fig1.tif
The crystal structure of Hg_2_SO_4_ in a projection along [010]. Displacement ellipsoids are drawn at the 74% probability level; short Hg—O bonds are displayed with closed black lines, longer Hg—O bonds with open lines.

CCDC reference: 1004277

Additional supporting information:  crystallographic information; 3D view; checkCIF report

## Figures and Tables

**Table:** 

Hg—O2^i^	2.193 (6)
Hg—O2^ii^	2.518 (6)
Hg—O1^iii^	2.725 (6)
Hg—O1^iv^	2.898 (7)
Hg—Hg^v^	2.5031 (7)
S—O1	1.450 (7)
S—O2	1.509 (6)

**Table:** 

O2^i^—Hg—Hg^v^	164.47 (14)

Symmetry codes: (i) 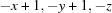; (ii) 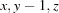; (iii) 
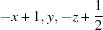
; (iv) 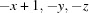; (v) 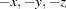.

## supplementary crystallographic information

### S1. Experimental

1 g HgO was suspended in 20 ml water. 4 ml sulfuric acid (96%_wt_) and 2 drops CS_2_ were added to the mixture, transferred into a 50 ml polypropylene beaker that was sealed and heated for 12 h at 393 K. Besides a polycrystalline dirty-white solid with an unknown diffraction pattern, few colourless and transparent single crystals of the title compound were present in the reaction mixture.

### S2. Refinement

The coordinates of the previous refinement (Dorm, 1969) were used as starting parameters. The highest and lowest remaining electron density is 0.84 Å and 1.25 Å, respectively, from the Hg atom. It should be noted that in the current version (01/2014) of the Inorganic Structure Data Base (ICSD, 2014), the deposited structure data of the previous refinement by Dorm (1969) contain an error: The *z* parameter of the sulfur atom must be 1/4, not 3/4.

### Crystal data

**Table:**

Hg_2_O_4_S	*F*(000) = 416
*M**_r_* = 497.24	*D*_x_ = 7.109 Mg m^−^^3^
Monoclinic, *P*2/*c*	Mo *K*α radiation, λ = 0.71073 Å
Hall symbol: -P 2yc	Cell parameters from 1233 reflections
*a* = 6.2771 (8) Å	θ = 3.3–30.4°
*b* = 4.4290 (6) Å	µ = 66.35 mm^−^^1^
*c* = 8.3596 (10) Å	*T* = 295 K
β = 91.695 (4)°	Fragment, colourless
*V* = 232.31 (5) Å^3^	0.18 × 0.08 × 0.04 mm
*Z* = 2	

### Data collection

**Table:**

Bruker SMART CCD diffractometer	701 independent reflections
Radiation source: fine-focus sealed tube	629 reflections with *I* > 2σ(*I*)'
Graphite monochromator	*R*_int_ = 0.060
ω–scans	θ_max_ = 30.4°, θ_min_ = 4.6°
Absorption correction: numerical (*HABITUS*; Herrendorf, 1997)	*h* = −8→8
*T*_min_ = 0.012, *T*_max_ = 0.119	*k* = −6→6
1737 measured reflections	*l* = −11→8

### Refinement

**Table:**

Refinement on *F*^2^	Primary atom site location: isomorphous structure methods
Least-squares matrix: full	*w* = 1/[σ^2^(*F*_o_^2^) + (0.052*P*)^2^ + 1.7115*P*] where *P* = (*F*_o_^2^ + 2*F*_c_^2^)/3
*R*[*F*^2^ > 2σ(*F*^2^)] = 0.035	(Δ/σ)_max_ < 0.001
*wR*(*F*^2^) = 0.087	Δρ_max_ = 3.57 e Å^−^^3^
*S* = 1.06	Δρ_min_ = −3.18 e Å^−^^3^
701 reflections	Extinction correction: *SHELXL*, Fc^*^=kFc[1+0.001xFc^2^λ^3^/sin(2θ)]^-1/4^
34 parameters	Extinction coefficient: 0.0118 (12)
0 restraints	

### Special details

**Table:**

Geometry. All e.s.d.'s (except the e.s.d. in the dihedral angle between two l.s. planes) are estimated using the full covariance matrix. The cell e.s.d.'s are taken into account individually in the estimation of e.s.d.'s in distances, angles and torsion angles; correlations between e.s.d.'s in cell parameters are only used when they are defined by crystal symmetry. An approximate (isotropic) treatment of cell e.s.d.'s is used for estimating e.s.d.'s involving l.s. planes.
Refinement. Refinement of *F*^2^ against ALL reflections. The weighted *R*-factor *wR* and goodness of fit *S* are based on *F*^2^, conventional *R*-factors *R* are based on *F*, with *F* set to zero for negative *F*^2^. The threshold expression of *F*^2^ > σ(*F*^2^) is used only for calculating *R*-factors(gt) *etc*. and is not relevant to the choice of reflections for refinement. *R*-factors based on *F*^2^ are statistically about twice as large as those based on *F*, and *R*- factors based on ALL data will be even larger.

### Fractional atomic coordinates and isotropic or equivalent isotropic displacement parameters (Å^2^)

**Table:**

	*x*	*y*	*z*	*U*_iso_*/*U*_eq_	
Hg	0.19318 (5)	0.05289 (9)	−0.02034 (4)	0.0275 (2)	
S	0.5000	0.5674 (5)	0.2500	0.0134 (5)	
O1	0.6943 (11)	0.3901 (16)	0.2586 (8)	0.0224 (12)	
O2	0.5038 (9)	0.7720 (13)	0.1058 (6)	0.0172 (10)	

### Atomic displacement parameters (Å^2^)

**Table:**

	*U*^11^	*U*^22^	*U*^33^	*U*^12^	*U*^13^	*U*^23^
Hg	0.0119 (2)	0.0392 (3)	0.0315 (3)	−0.00369 (11)	0.00211 (13)	0.00072 (13)
S	0.0125 (12)	0.0141 (11)	0.0136 (11)	0.000	0.0000 (8)	0.000
O1	0.018 (3)	0.027 (3)	0.022 (3)	0.009 (2)	−0.001 (2)	0.004 (2)
O2	0.015 (2)	0.020 (2)	0.016 (2)	0.001 (2)	0.0010 (18)	0.004 (2)

### Geometric parameters (Å, º)

**Table:**

Hg—O2^i^	2.193 (6)	S—Hg^iii^	3.7082 (15)
Hg—O2^ii^	2.518 (6)	S—Hg^ix^	3.8936 (17)
Hg—O1^iii^	2.725 (6)	S—Hg^iv^	3.8936 (17)
Hg—O1^iv^	2.898 (7)	O1—Hg^iii^	2.725 (6)
Hg—Hg^v^	2.5031 (7)	O1—Hg^iv^	2.898 (7)
S—O1^iii^	1.450 (7)	O1—Hg^i^	3.261 (7)
S—O1	1.450 (7)	O1—Hg^vii^	3.716 (7)
S—O2^iii^	1.509 (6)	O1—Hg^x^	4.090 (6)
S—O2	1.509 (6)	O2—Hg^i^	2.193 (6)
S—Hg^vi^	3.2315 (13)	O2—Hg^viii^	2.518 (6)
S—Hg^i^	3.2315 (13)	O2—Hg^vi^	3.812 (5)
S—Hg^vii^	3.6309 (15)	O2—Hg^vii^	4.097 (6)
S—Hg^viii^	3.6309 (15)		
			
O2^i^—Hg—Hg^v^	164.47 (14)	S—O1—Hg^x^	156.0 (3)
O2^i^—Hg—O2^ii^	69.1 (2)	Hg^iii^—O1—Hg^x^	36.63 (8)
Hg^v^—Hg—O2^ii^	126.30 (13)	Hg^iv^—O1—Hg^x^	77.69 (14)
O2^i^—Hg—O1^iii^	82.0 (2)	Hg^i^—O1—Hg^x^	117.52 (19)
Hg^v^—Hg—O1^iii^	102.86 (14)	Hg^vii^—O1—Hg^x^	89.04 (14)
O2^ii^—Hg—O1^iii^	75.8 (2)	S—O1—Hg	62.8 (3)
O2^i^—Hg—O1^iv^	77.5 (2)	Hg^iii^—O1—Hg	115.4 (2)
Hg^v^—Hg—O1^iv^	102.91 (14)	Hg^iv^—O1—Hg	64.22 (13)
O2^ii^—Hg—O1^iv^	75.64 (18)	Hg^i^—O1—Hg	95.95 (15)
O1^iii^—Hg—O1^iv^	149.3 (3)	Hg^vii^—O1—Hg	138.14 (18)
O1^iii^—S—O1	114.5 (6)	Hg^x^—O1—Hg	129.82 (18)
O1^iii^—S—O2^iii^	109.4 (3)	S—O2—Hg^i^	120.5 (3)
O1—S—O2^iii^	108.6 (4)	S—O2—Hg^viii^	126.9 (3)
O1^iii^—S—O2	108.6 (4)	Hg^i^—O2—Hg^viii^	110.9 (2)
O1—S—O2	109.4 (3)	S—O2—Hg^vi^	56.41 (19)
O2^iii^—S—O2	106.2 (5)	Hg^i^—O2—Hg^vi^	131.7 (2)
S—O1—Hg^iii^	122.3 (4)	Hg^viii^—O2—Hg^vi^	80.47 (14)
S—O1—Hg^iv^	123.6 (4)	S—O2—Hg	72.7 (2)
Hg^iii^—O1—Hg^iv^	96.8 (2)	Hg^i^—O2—Hg	129.6 (2)
S—O1—Hg^i^	76.0 (3)	Hg^viii^—O2—Hg	85.11 (15)
Hg^iii^—O1—Hg^i^	148.1 (3)	Hg^vi^—O2—Hg	97.20 (13)
Hg^iv^—O1—Hg^i^	91.77 (18)	S—O2—Hg^vii^	61.6 (2)
S—O1—Hg^vii^	75.3 (3)	Hg^i^—O2—Hg^vii^	77.46 (15)
Hg^iii^—O1—Hg^vii^	85.41 (17)	Hg^viii^—O2—Hg^vii^	122.59 (19)
Hg^iv^—O1—Hg^vii^	153.3 (2)	Hg^vi^—O2—Hg^vii^	58.72 (8)
Hg^i^—O1—Hg^vii^	73.82 (14)	Hg—O2—Hg^vii^	134.35 (14)

Symmetry codes: (i) −*x*+1, −*y*+1, −*z*; (ii) *x*, *y*−1, *z*; (iii) −*x*+1, *y*, −*z*+1/2; (iv) −*x*+1, −*y*, −*z*; (v) −*x*, −*y*, −*z*; (vi) *x*, −*y*+1, *z*+1/2; (vii) −*x*+1, *y*+1, −*z*+1/2; (viii) *x*, *y*+1, *z*; (ix) *x*, −*y*, *z*+1/2; (x) *x*+1, −*y*, *z*+1/2.
