# Supplementary figures and images for: Crystal structure of Hg2SO4 – a redetermination
Source: Acta Crystallogr Sect E Struct Rep Online. 2014 Aug 1;70(Pt 9):i44. doi: 10.1107/S1600536814011155 (PMC4186147; doi:10.1107/S1600536814011155)

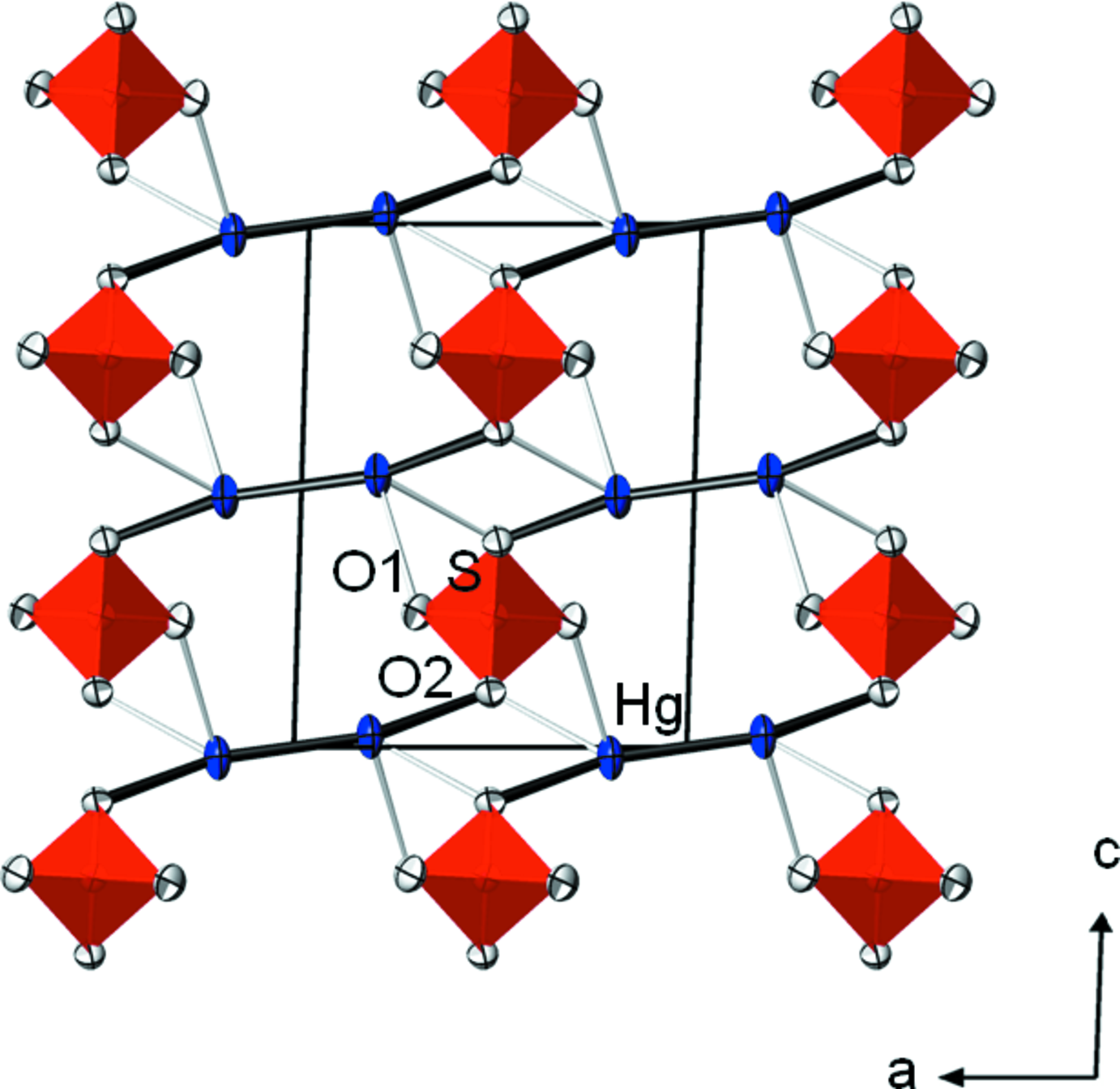

Supplement: Supplementary file 3 [file e-70-00i44-fig1.tif]
